# Impact of non-invasive mechanical ventilation (niv) in critical patients with influenza (H1N1) PDM09

**DOI:** 10.1186/2197-425X-3-S1-A702

**Published:** 2015-10-01

**Authors:** C Ferri, J Marin-Corral, M Magret, M Bodí, S Trefler, E Díaz, I Martín-Loeches, JC Yébenes, C Cilloniz, J Masclans, F Gordo-Vidal, L Cordero, A Rodríguez

**Affiliations:** Critical Care Department, Hospital Universitari Joan XXIII de Tarragona, Tarragona, Spain; CIBERES (CIBER Enfermedades Respiratorias), Palma de Mallorca, Spain; Critical Care Department, Parc Taulí Hospital, Sabadell, Spain; Department of Anaesthesia and Critical Care, Multidisciplinary Intensive Care Research Organization (MICRO). St. James university Hospital. Trinity Centre For Health Sciencies, Dublin, Ireland; Critical Care Department, Hospital de Mataró, Mataró, Spain; Hospital Clinic de Barcelona, Critical Care Department, Barcelona, Spain; Hospital del Mar, Critical Care Department, Barcelona, Spain; Hospital de Henares, Critical Care Department, Madrid, Spain; CHUAC, Critical Care Department, A Coruña, Spain

## Introduction

The use of non-invasive mechanical ventilation (NIV) in patients with influenza A (H1N1)pdm09 admitted to intensive care units (ICU) has been controversial.

## Objectives

Our objective was to assess the incidence of failure in NIV in this group of patients and their impact on ICU mortality rate.

## Methods

Secondary analysis of prospective observational multicentric study in 148 spanish ICUs. Data was obtained of GTEI / SEMICYUC (2009-2014) registry. All patients with Influenza Virus A (H1N1) confirmed with rt-PCR were included. Ventilatory strategy, demographics and hemodynamic data, comorbidities and severity indexes were evaluated and they were correlated with mortality. Chi-square (categorical variables) and “t” test or Mann-Whitney test (continuous variables) analysis were performed. Significant variables in the univariate analysis were included in a multivariate model (conditional logistic regression). A “p” value less than 0.05 was considered significant.

## Results

2.223 patients were included in the analysis with a mortality 21.1% (n = 470 patients). 1726 patients were ventilated (77.6%), 962 (55.7%) of them were initially intubated, and in 764 (44.3%) NIV was initiated. NIV failed in 464 (60.7%) while 300 patients were responders (39.3%). Patients who died presented: older age (53.5 [15.34] vs. 48.5[15.1], p = 0.000), predominantly male (65% vs. 35%, p = 0.000), higher APACHEII (21[8] vs.14[6], p = 0.000) and SOFA (8[3] vs. 5[3], p = 0.000), more shock (79% vs. 44%, p < 0.000), more acute renal failure (49% vs. 18%, p = 0.000), more comorbidities (asthma, heart failure, renal failure and immunosuppression, p < 0.001), more days of mechanical ventilation (12.9[13.4]vs.9.4[13.2], p = 0.000) and longer hospital stay (23.2[19.3]vs. 16.6[15.6]p = 0.000). NIV failed group patients, had higher mortality (36%) than NIV successful group (4%, p = 0.000) and initially intubated group (31%, p = 0.07). Furthermore, the failure of NIV (OR=10.2, 95%IC 5.28-19.76, p = 0.000), the APACHEII (OR=1,05, 95%IC 1.02-1.09, p = 0.004), acute renal failure (OR=2.48, 95% IC 1.52-4.05, p = 0.000) and immunodeficiency (OR=5.66, 95%IC 3.02-10.60, p = 0.000) were independently variables associated with mortality in the multivariate analysis.

## Conclusions

In our population of patients with influenza A (H1N1)pdm09, the failure of NVI is frequently and is associate independently with the ICU mortality.
